# A Machine Learning Model Based on Radiomic Features as a Tool to Identify Active Giant Cell Arteritis on [^18^F]FDG-PET Images During Follow-Up

**DOI:** 10.3390/diagnostics15030367

**Published:** 2025-02-04

**Authors:** Hanne S. Vries, Gijs D. van Praagh, Pieter H. Nienhuis, Lejla Alic, Riemer H. J. A. Slart

**Affiliations:** 1 Department of Nuclear Medicine and Molecular Imaging, University Medical Centre Groningen, University of Groningen, 9700 RB Groningen, The Netherlands; 2 Department of Magnetic Detection & Imaging, Technical Medical Centre, Faculty of Science and Technology, University of Twente, 7522 NH Enschede, The Netherlands

**Keywords:** giant cell arteritis, atherosclerosis, [^18^F]FDG PET, therapy monitoring, radiomics

## Abstract

**Objective**: To investigate the feasibility of a machine learning (ML) model based on radiomic features to identify active giant cell arteritis (GCA) in the aorta and differentiate it from atherosclerosis in follow-up [^18^F]FDG-PET/CT images for therapy monitoring. **Methods**: To train the ML model, 64 [^18^F]FDG-PET scans of 34 patients with proven GCA and 34 control subjects with type 2 diabetes mellitus were retrospectively included. The aorta was delineated into the ascending, arch, descending, and abdominal aorta. From each segment, 95 features were extracted. All segments were randomly split into a training/validation (*n* = 192; 80%) and test set (*n* = 46; 20%). In total, 441 ML models were trained, using combinations of seven feature selection methods, seven classifiers, and nine different numbers of features. The performance was assessed by area under the curve (AUC). The best performing ML model was compared to the clinical report of nuclear medicine physicians in 19 follow-up scans (7 active GCA, 12 inactive GCA). For explainability, an occlusion map was created to illustrate the important regions of the aorta for the decision of the ML model. **Results**: The ten-feature model with ANOVA as the feature selector and random forest classifier demonstrated the highest performance (AUC = 0.92 ± 0.01). Compared with the clinical report, this model showed a higher PPV (0.83 vs. 0.80), NPV (0.85 vs. 0.79), and accuracy (0.84 vs. 0.79) in the detection of active GCA in follow-up scans. **Conclusions**: The current radiomics ML model was able to identify active GCA and differentiate GCA from atherosclerosis in follow-up [^18^F]FDG-PET/CT scans. This demonstrates the potential of the ML model as a monitoring tool in challenging [^18^F]FDG-PET scans of GCA patients.

## 1. Introduction

Giant cell arteritis (GCA) is a systemic inflammatory condition primarily affecting the vessel walls of the aorta, its main branches, and the arteries in the head and neck region [1]. This inflammation can lead to arterial stenosis and weakening of the aortic wall, which can cause ischemia in downstream tissue and aortic aneurysm formation. Accurate diagnosis is essential to prevent severe outcomes, including vision loss or fatal complications [2,3]. However, diagnosing GCA is challenging due to its often-nonspecific symptoms. Currently, the diagnosis relies strongly on clinical findings and various imaging modalities, i.e., computed tomography (CT), magnetic resonance imaging, ultrasound, and 2-deoxy-2-[^18^F]fluoro-D-glucose positron emission tomography ([^18^F]FDG-PET) [4]. However, each of these imaging techniques has its unique limitations. While CT can effectively detect calcified plaques, it struggles with identifying non-calcified, fatty plaques, and has difficulty distinguishing atherosclerosis from vasculitis, which can lead to misdiagnosis. MRI, although non-invasive, is not typically used as a primary imaging modality for GCA due to limited evidence supporting its effectiveness in visualising vascular inflammation [5]. Due to the increased glycolytic activity of activated inflammatory cells, [^18^F]FDG serves as a potential indicator of inflammation [6]. [^18^F]FDG PET/CT has a high diagnostic sensitivity and specificity for GCA [7,8]; however, its ability to distinguish active disease from remission in patients during treatment is moderately effective [9].

As GCA is characterised by granulomatous inflammation with the presence of giant cells, this inflammation results in a homogeneous pattern of [^18^F]FDG uptake along vessel walls [10]. In contrast, atherosclerosis, a chronic inflammatory disease, presents with a patchier and heterogeneous [^18^F]FDG uptake due to lipid-rich plaques and calcifications [11]. In their own respective ways, both are inflammatory conditions causing arterial damage or narrowing. Consequently, patients are at risk of aneurysm formation or end-organ ischemia. Despite the differences, both conditions exhibit elevated [^18^F]FDG uptake, which complicates their visual differentiation. This is especially challenging in follow-up scans of patients during and after treatment, where nuclear medicine physicians (NMPs) often face difficulties in distinguishing between the two conditions. Given the substantial side effects of the high-dose glucocorticoid treatment for GCA, treatment stratification is crucial [2]. Therefore, the correct identification of active GCA is of paramount importance [5].

Image-based texture features are increasingly being investigated as surrogate imaging biomarkers for the quantitative assessment of [^18^F]FDG-PET uptake as a proxy for underlying biological processes [12,13]. This analysis, referred to as radiomics, contains information that may not be appreciated by the human eye [14]. Radiomics in PET-based vascular imaging has proven to offer valuable insights for plaque characterisation and the diagnosis of aortic GCA [15,16]. Despite the promising research findings, the clinical application of radiomics is yet to be confirmed. The development of machine learning (ML) models offers an opportunity to enhance the extraction of meaningful information from PET images, potentially assisting NMPs in their diagnostic decisions [17].

Therefore, the aim of this study is to investigate the feasibility of an ML model based on radiomic features to identify active GCA in the aorta in follow-up [^18^F]FDG-PET images. Objectives in achieving this goal were twofold: first, to investigate the feasibility of an ML model to distinguish GCA from atherosclerosis in aortic [^18^F]FDG-PET images; second, to assess the clinical efficacy of the ML model in detecting active GCA in follow-up [^18^F]FDG-PET images compared to the current standard of care provided by the visual assessment of nuclear medicine physicians.

## 2. Materials and Methods

### 2.1. Data

#### 2.1.1. Patient and Study Protocol

This study included 87 [^18^F]FDG-PET/CT scans from 76 patients, along with corresponding clinical data, collected between 2011 and 2020 as part of two prospective cohorts. Both protocols were reviewed and approved by the Medical Ethical Institutional Review Board of the University Medical Center Groningen (UMCG, number 2010-222 (GCA/PMR/SENEX (GPS) cohort [18]) and 2013-080 (RELEASE cohort [19]). All participants gave written informed consent, and the studies were conducted in compliance with the principles of the Declaration of Helsinki.

Fifty-three [^18^F]FDG-PET/CT scans were from patients with proven GCA from the GPS cohort, consisting of 34 baseline [^18^F]FDG-PET/CT scans from 34 patients (GCA group 1) and 19 follow-up [^18^F]FDG-PET/CT scans from 15 patients (GCA group 2). Baseline scans were included if (i) the primary GCA diagnosis remained unchanged for at least six months after the first symptoms; (ii) an [^18^F]FDG-PET/CT scan was performed at the time of diagnosis. Patients were excluded if they received more than three days of (high-dose) prednisone therapy at the time of the [^18^F]FDG-PET/CT scan, as prednisone is known to reduce [^18^F]FDG uptake [20]. The follow-up scans (GCA group 2) were acquired at least six months after GCA diagnosis. This group was subdivided into seven scans from seven patients with active GCA (GCA group 2a) and twelve scans from eleven patients in whom GCA was inactive (GCA group 2b). Active disease was defined as a recurrence of clinical disease activity after a period of remission, necessitating an increase in medical drug intake or additional medical drug prescription, after the evaluation of the clinical presentation and the [^18^F]FDG-PET scan [21]. GCA was defined as inactive when the medical drug level stayed the same or was decreased [21]. Seven patients (with eight scans) from GCA group 2 were also in GCA group 1.

Thirty-four control patients with type 2 diabetes mellitus (atherosclerosis group) were included from the RELEASE cohort. The inclusion and exclusion criteria of the atherosclerosis group were discussed before [19], but briefly: the patients diagnosed with type 2 diabetes mellitus were included if (i) they had an assessable pulse wave velocity at screening; (ii) were on a stable dose of medication to regulate blood pressure—and/or lipid content. Exclusion criteria were as follows: (i) the current use of glucose-lowering drugs; (ii) diagnosis of cardiovascular disease; (iii) uncontrolled hypertension. Individuals with type 2 diabetes mellitus have in general a high formation of calcifications (i.e., atherosclerosis) [22]. In the RELEASE cohort, other vascular diseases were ruled out [19]. Therefore, vascular [^18^F]FDG uptake was assumed to be related to atherosclerosis.

**Figure 1:**
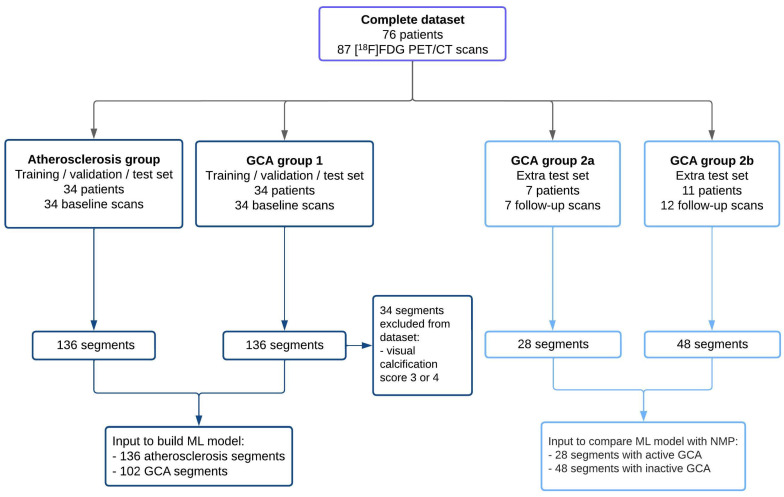
Dataset flowchart for atherosclerosis group, GCA group 1 and GCA group 2. GCA = giant cell arteritis; ML = machine learning; NMP = nuclear medicine physician.

GCA group 1 and the atherosclerosis group were used for the development of the ML model (training, validation, and test), and GCA group 2 was used for the assessment of clinical efficacy in follow-up scans (extra test). See Table 1 for all patient characteristics and Figure 1**.** for a flowchart of the complete dataset.

#### 2.1.2. Image Acquisition and Reconstruction

All [^18^F]FDG-PET/CT scans were acquired using an integrated PET/CT system (Biograph mCT 40 or 64-slice, Siemens Healthineers, Knoxville, TN, USA) according to the European Association of Nuclear Medicine (EANM) procedure guidelines for imaging following EARL criteria [23]. All subjects fasted for at least six hours prior to [^18^F]FDG injection (3 MBq/kg). The images were acquired 60 min post-injection from skull to knee, with 2–3 min per table position. The voxel size for [^18^F]FDG-PET was 3.19 × 3.19 × 2 mm^3^. Prior to the PET, a low-dose CT was acquired for attenuation correction and anatomic localisation. See Table 1 for the patient characteristics and PET/CT acquisition and reconstruction parameters.

#### 2.1.3. Segmentation

The aortas were manually delineated in the low-dose CT images into four segments (the ascending aorta, aortic arch, descending aorta, and abdominal aorta) using Affinity Viewer (version 2.0.3; Hermes Medical Solutions, Stockholm, Sweden). This segmentation was utilised by overlaying the co-registered PET image and was used to ensure the inclusion of all arterial [^18^F]FDG uptake while excluding any spill-over uptake from neighbouring tissue. Co-registration of the PET and CT images was achieved using the integrated hybrid PET/CT systems, utilising a post-processing step provided by the manufacturer. Alignment was based on the system’s bed position, ensuring precise image registration without the need for additional rigid or deformable registration methods. This approach facilitated accurate segmentation in the CT scans. All annotations were conducted by two trained observers with over three years of experience in the field.

#### 2.1.4. Exclusion of Calcified Segments in GCA Group

To ensure the development of an ML model capable of accurately differentiating between atherosclerosis and GCA, we aimed to create a training dataset that was as refined as possible. Given the potential confounding effect of atherosclerotic [^18^F]FDG uptake patterns on the identification of GCA-related uptake patterns, we conducted a pre-selection of aortic segments within GCA group 1.

Specifically, aortic segments with significant calcification were excluded from GCA group 1. Calcifications were assessed visually on the low-dose CT using Rominger’s method [24]. Segments presenting with a calcification score of 3 or 4—indicating more than 25% of the vessel wall was calcified—were excluded from GCA group 1. The Supplementary Methods (Part A) include an example of the calcification grading. In total, 34 segments were excluded, resulting in a total of 102 aortic segments for GCA group 1 (Figure 1). By eliminating these segments, we intended to reduce the likelihood of atherosclerosis-related [^18^F]FDG uptake confounding the training process for GCA identification.

To represent a clinical situation, no calcification grading or segment exclusion was performed in GCA group 2.

### 2.2. Machine Learning Model

#### 2.2.1. Pipeline

All PET images were converted from activity (Bq/mL) into standardised uptake values normalised to lean body mass (LBM), referred to as *SUL*, as recommended for vasculitis studies [25]. The *SUL* is calculated by the following equation:

(1) $$SUL=\frac{\;Tissue\;activity\;}{\frac{Injected\;dose}{LBM}}$$

A sex-specific LBM was used:

(2) $${LBM}_{male}=9270\times \frac{body\;weight}{6680+216\times BMI}$$

(3) $${LBM}_{female}=9270\times \frac{body\;weight}{8780+244\times BMI}$$

All PET images were resampled to isotropic voxel spacing of 2.0 × 2.0 × 2.0 mm^3^ using cubic B-Spline interpolation. A total of 93 radiomics features were extracted: 18 FOS features, 24 GLCM features, 16 GLRLM features, 16 GLSZM features, 5 NGRDM features, and 14 GLDM features. In addition to the 93 radiomics features, 2 additional quantitative features were also extracted: SULmax and SULmean, making a total of 95 features. Features were extracted using the standardised framework for radiomics in python, pyRadiomics, which is in compliance with the Image Biomarker Standardisation Initiative (IBSI) guidelines [26]. Features with a high linear correlation (Pearson correlation coefficient (Pearson’s r) above 0.9) were removed from the dataset.

To find the most optimal ML model, we trained and validated 441 different models using combinations of seven feature selection methods, nine different numbers of features, and seven classifiers. The feature selection methods and classifiers were chosen for their popularity in the literature, computational efficiency, and publicly available implementations, which increases reusability [27]. A detailed explanation of the feature extraction, the list of the extracted features, feature selection methods, and classifiers can be found in the Supplementary Methods—part B.

For the development of the ML model, the baseline dataset (GCA group 1 and atherosclerosis group) was randomly split into training/validation (80%; *n* = 192 segments) and hold-out test set (20%; *n* = 46 segments). The dataset was split patient-wise, so all segments of one patient were assigned to either the training or test set. All ML models were trained using ten-fold cross-validation. All steps in feature selection and classification were executed using the Sci-kit Learn package (version 1.1.3) in Python (version 3.9.12).

#### 2.2.2. Performance Final Model—Test Set

The performance of all models was evaluated by the area under the receiver operating characteristic curve (AUC), positive prediction value (PPV), and negative prediction value (NPV) [28]. The most optimal model in the validation set was chosen using a combination of the AUC, PPV, and NPV. This model, hereafter referred to as ‘final model’, was then applied to the hold-out test set to assess its performance. Additionally, the final model was evaluated on the extra test set.

#### 2.2.3. ML Model Features and Occlusion Map

To ensure the ML model is interpretable and can be effectively used a supportive tool in clinical practise, we analysed the key features of the final model by examining their importance weights. Additionally, we generated occlusion maps to identify specific regions of the aorta that most significantly influenced the model’s final decision. The maps were generated by systematically occluding different regions of the aorta and the resulting changes were measured in their prediction probability [29,30,31]. This information is converted into a heatmap to highlight the aortic locations for the detection of active GCA. A technical explanation of the occlusion map is provided in the Supplementary Methods—Part C.

#### 2.2.4. Performance Final Model—Extra Test Set (Follow-Up)

The findings/conclusions of the nuclear medicine physicians were retrospectively retrieved from the electronic patient file system and analysed. The outcomes were divided into inactive GCA (nuclear medicine physician reported no or uncertain active aortic uptake) and active GCA (nuclear medicine physician reported active aortic uptake).

Then, the [^18^F]FDG PET scans from GCA group 2 were fed into the final model as an extra test set. The highest predicted probability among the four segments was chosen as the outcome of the final model. For example, if the ascending aorta has a probability of 0.45, the descending aorta 0.75, the aortic arch 0.66, and the abdominal aorta 0.74, the probability of this scan will be 0.75. An ROC analysis was performed, and the optimal cut-off point was estimated using the generalised Youden’s index.

The reports of the nuclear medicine physicians and the outcomes of the final model were compared with the final diagnosis as described in the ‘patient and study protocol’ section. Performance was reported as accuracy, PPV, and NPV.

### 2.3. Statistical Analysis

After testing for normality, the baseline patient characteristics were compared using the independent student’s *t*-test or the Mann–Whitney U test. Continuous variables are displayed as the mean with standard deviation (mean ± SD) or median with interquartile range (median [IQR]). Categorial variables are displayed as numbers (percentages). A *p* < 0.05 was considered statistically significant. The statistical analyses were performed using the Statistical Package for the Social Sciences (version 27.0, SPSS Inc., Chicago, IL, USA).

## 3. Results

### 3.1. Patient Characteristics

The patient characteristics of the baseline set and follow-up set are summarised in Table 1. The mean age of the GCA group 1 and atherosclerosis group were 70 ± 8.1 years and 61 ± 8.7 years (*p* < 0.001), respectively. Furthermore, differences were found between body mass index (*p* < 0.001) and glucose levels (*p* = 0.003). There were no significant differences between GCA group 2a and GCA group 2b. The patient characteristics of the follow-up scans were presented per scan instead of per patient, as some patients had two follow-up scans.

### 3.2. Machine Learning Model

#### 3.2.1. Performance Final Model—Test Set

**Figure 2:**
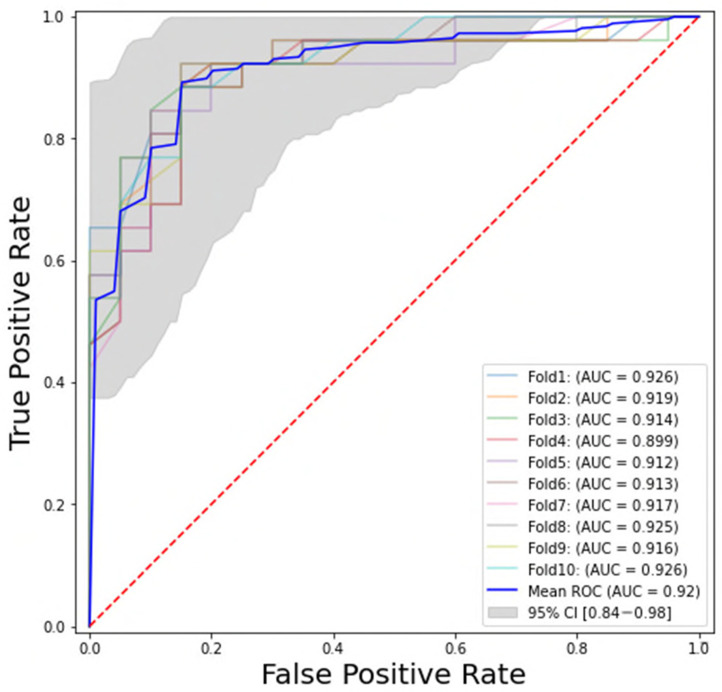
The receiver operating characteristic (ROC) curve of the final model, ANOVA feature selection method, and the RF classifier with ten features. All colours represent the ROC curve of a fold. The dark blue curve is the mean of all ten folds. The grey area is the 95% confidence interval of the 10 folds.

Of the 95 extracted features, 46 features had a Pearson’s r value higher than 0.9 and were removed from the dataset. Among all 441 combinations, the ten-feature model with ANOVA feature selection and a random forest classifier was selected as the final model. The performance of this model was calculated on the test set, showing the following values: AUC = 0.92 ± 0.01, PPV = 0.89 ± 0.04, and NPV = 0.82 ± 0.05. The ROC curve of this model is shown in Figure 2.

#### 3.2.2. Performance Final Model—Extra Test Set (Follow-Up)

Next, the final model was tested on GCA groups 2a and 2b. Using Youden’s index, the optimal cut-off point for the final model was 0.90. Based on this cut-off point, the final model scored higher on accuracy (0.84 vs. 0.79), PVV (0.83 vs. 0.80), and NPV (0.85 vs. 0.79) compared with the reports of the nuclear medicine physicians (Table 2).

#### 3.2.3. ML Model Features and Occlusion Map

**Figure 3:**
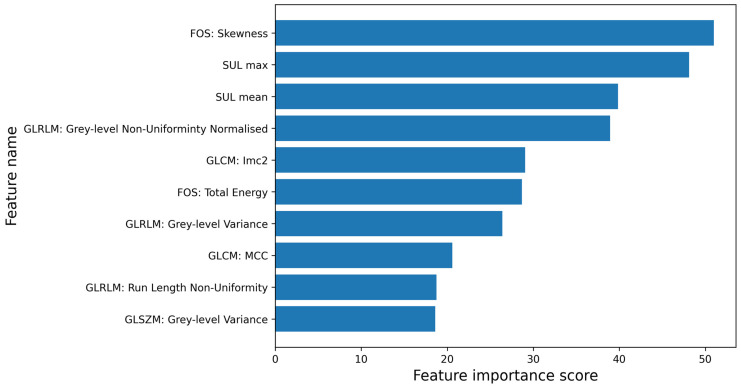
Feature importance of final model with ANOVA feature selection method and RF classifier using ten features, showing FOS skewness as most important feature.

The feature importance of the final model is illustrated in Figure 3. The final model based its outcome on four intensity features (including SULmean and SULmax) and six texture features. The four intensity values showed significantly higher values for the GCA group (*p* < 0.001). The normalised GLRLM grey-level non-uniformity and GLCM lmc2 were the most important texture features. The GLRLM feature showed lower values for the GCA group (*p* < 0.001), whereas the GLCM feature showed higher values for the GCA group (*p* < 0.001).

**Figure 4:**
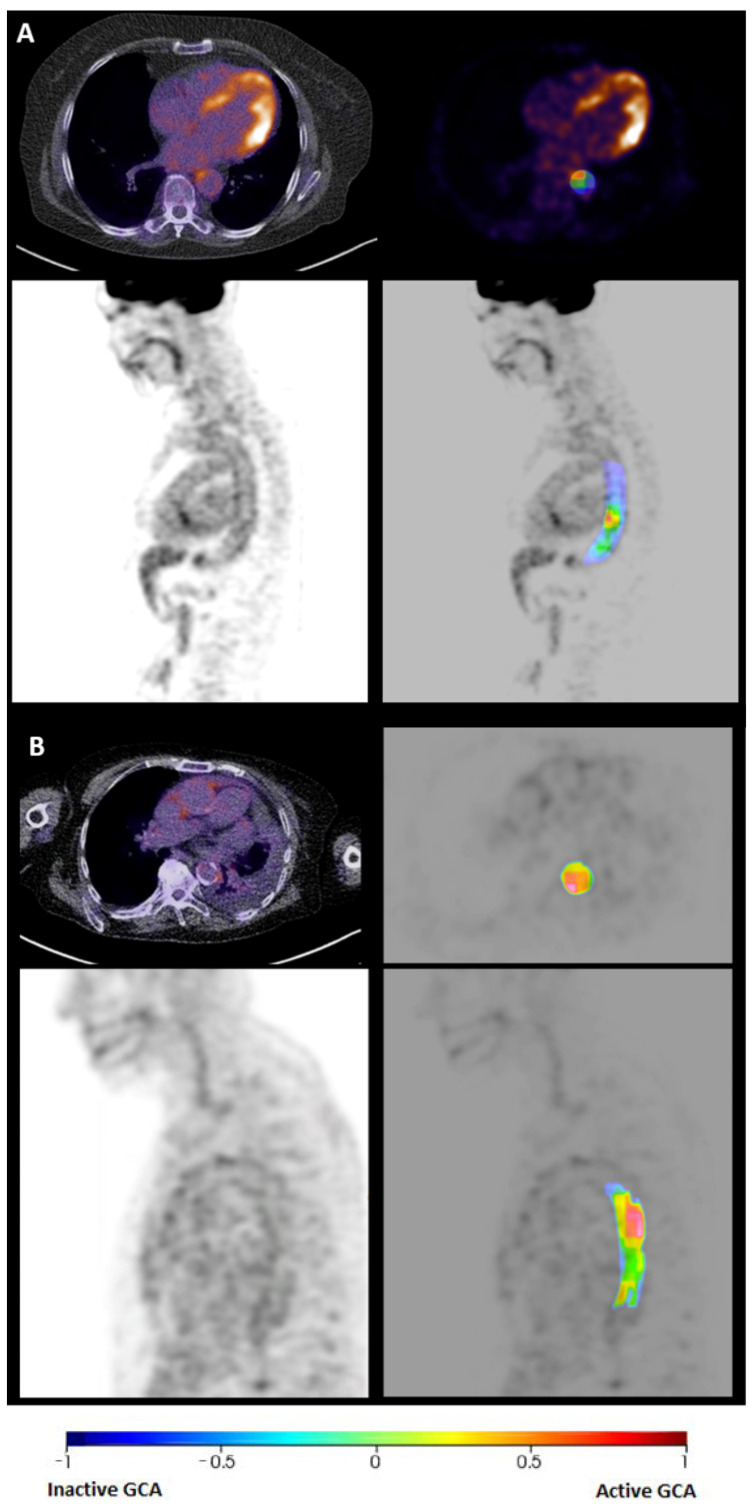
An occlusion map of two different follow-up scans illustrating the relative importance of different regions in the decision-making of the final model. The bar shows the difference in probability when that part of the image is occluded compared to the final probability of the model when no parts are occluded. Regions with a deeper red colour indicate a stronger contribution to the model’s prediction of active GCA, while regions with a deeper blue colour indicate a greater contribution to the model’s prediction of inactive GCA. (**A**) Descending aorta of inactive GCA; (**B**) descending aorta of active GCA.

Moreover, Figure 4 demonstrates two examples of the developed occlusion maps of the aorta. The red regions show the regions with a relatively high contribution to active GCA, and the blue regions show the regions with a relatively higher contribution to inactive GCA.

## 4. Discussion

In this proof-of-concept study, we explored the potential of an ML model to identify active GCA in the aorta in follow-up [^18^F]FDG-PET scans. This was achieved by training an ML model on distinguishing GCA from atherosclerosis. The model demonstrated high performance, surpassing the accuracy of nuclear medicine physicians’ reports. This demonstrates its potential as a valuable tool for the evaluation of GCA activity. Furthermore, to enhance the usability of the ML model’s decision-making process, an occlusion map was generated to highlight the important regions of the aorta.

Accurate and fast diagnosis of GCA is crucial due to the need for high-dose glucocorticoid treatment, which can lead to severe side effects [3]. Although [^18^F]FDG-PET is widely used for diagnosis, distinguishing active GCA from inflammatory atherosclerotic processes can complicate visual diagnosis, particularly in follow-up scans where glucocorticoid treatment reduces uptake [2,32]. The developed ML model could become a valuable clinical tool to assist the nuclear medicine physician in detecting active GCA, and act as a “co-reader”.

Radiomic features extracted from PET images provide detailed spatial information about voxel intensity distribution within the image, allowing for the characterisation of [^18^F]FDG uptake at a higher spatial resolution than is visible to the human eye [33]. In our ML model, the radiomic features with the highest feature importance were intensity features (FOS skewness and SULmax). FOS features describe the intensity distribution of voxels within the volume of interest [26]. For all intensity features, GCA exhibited significantly higher values than atherosclerosis, indicating increased [^18^F]FDG uptake [26]. These findings align with studies that report higher intensity values in GCA compared to atherosclerosis [34,35,36]. Espitia et al. compared [^18^F]FDG-PET uptake in GCA and aortic atheroma and found a significantly higher SUVmax, ΔSUV, and target-to-background ratio in the GCA group [37]. Similarly, our results showed increased SULmax and SULmean values in the GCA group.

The ML model also incorporated features describing the heterogeneity of the uptake pattern, specifically GLRLM and GLCM features [38]. Higher GLRLM feature values indicate a more heterogeneous texture pattern [26]. Since the visual appearance of GCA is more homogeneous than atherosclerosis, the significantly lower GLRLM values for the GCA group align with findings in the literature [11,15,37]. For the GLCM feature, values closer to one indicate more dependent and uniform voxel distributions. The GCA group showed higher values, indicating a homogeneous uptake pattern, which is also in accordance with the literature [11]. In this proof-of-concept study, only radiomics from PET images were included. Future studies could explore adding radiomics from diagnostic CT images, as combining PET and CT radiomic features has demonstrated improvements in a similar study, albeit in another pathology [39].

Besides the feasibility of distinguishing between GCA and atherosclerosis, the final model showed a higher PPV, NPV, and accuracy than the clinical report based on the visual assessment of the nuclear medicine physician for identifying active GCA. This comparison underscores the potential of the model to improve diagnostic accuracy beyond traditional visual evaluation of imaging scans. In addition to outperforming the visual assessment, the model achieved an overall accuracy of 0.84, demonstrating its potential to accurate identify active GCA. However, as noted by Hellmich et al., imaging findings from follow-up scans should be interpreted with caution. Elevated uptake does not necessarily indicate actual active disease but could also represent remodelling and should therefore be compared to previous imaging findings of the patient [4]. This highlights the importance of using the ML model as a complement to, rather than a replacement for, nuclear medicine physicians.

Discussion about the adoption of ML models in clinical practise is growing. This is partly due to the ‘black-box’ nature of these models, which makes it challenging for clinicians to understand how decisions are made [40,41]. To facilitate the integration of ML models into clinical practise, it is essential that clinical experts understand the decision-making process behind the model. One way to enhance interpretability is using of occlusion sensitivity maps, which visually highlight the regions of an image that most influence the model’s decision [31]. In this study, these maps provided a clear visual representation of the key aortic areas that influenced the ML model’s output. By identifying these crucial regions, the map can assist the nuclear medicine physician in confirming or refining their diagnostic assessments, ultimately leading to more confident and informed decision-making, particularly in complex cases. To fully evaluate the clinical benefit of combining ML models with an occlusion map, a prospective study should be conducted. Such a study would compare the diagnostic performance of nuclear medicine physicians using the ML model with occlusion maps to their performance without occlusion maps. This would help determine whether the combination enhances diagnostic confidence and accuracy in a clinical practise.

To the best of our knowledge, this is the first study training an ML model on a dataset containing both GCA and atherosclerosis cases, allowing us to differentiate between the two conditions based on differences in [^18^F]FDG uptake. Previous studies by Duff et al. used radiomics to diagnose GCA, reporting high performance, with AUC scores ranging from 0.80 to 1.00 [16,42]. However, their study focused solely on primary GCA, whereas our study demonstrates the feasibility of applying this model in challenging follow-up cases. Furthermore, Duff et al. excluded patients with primary GCA if imaging showed no evidence of active GCA and control subjects with imaging evidence of atherosclerosis-related activity in the aorta. This created a selection bias by excluding more complex cases. Several other studies have explored new scoring systems to distinguish between GCA and atherosclerosis [36], but none have specifically addressed this in follow-up patients. For example, Bacour et al. introduced a visual scoring approach to differentiate GCA and atherosclerosis in [^18^F]FDG PET [43]. Their objective was to develop a reliable, standardised visual scoring system, but their study included only patients with temporal artery biopsy-proven GCA and severe atherosclerosis, yielding high AUC values. While visual scoring is well known to be useful for diagnosing primary GCA, it is more challenging in follow-up. Furthermore, it is prone to observer variability. In contrast to Bacour et al., we used a quantitative, more objective method to differentiate between GCA and atherosclerotic [^18^F]FDG uptake. By evaluating our model on follow-up scans of treated patients, we address the most diagnostically challenging group, underlining the importance of this study.

Although our results are promising, there are opportunities to further improve the accuracy and applicability of the model. One potential direction is to introduce a more diverse dataset, including larger, multicentre cohorts. Testing performance with larger, multicentre cohorts would also be particularly beneficial for the application of deep learning methods, which thrive on greater data diversity and volume. Additionally, integrating clinical features into the training process could further refine the model’s predictive performance. Multimodal approaches that combine radiomic features from different imaging modalities and clinical data have shown significant improvements in different clinical contexts [39,44].

This study has some limitations. First, the study is a single-centre, single-scanner, retrospective study. Moreover, in radiomics studies, it is important to consider the impact of a limited sample size, as this can increase the risk of overfitting. Therefore, we used feature selection methods to reduce the dimensionality of the dataset and focused on relevant features. We also increased the input samples for the training of the model by dividing the aorta into four segments and tested the model on unseen data to evaluate the performance. While these steps reduce the likelihood of overfitting, they do not eliminate it entirely. In future studies, it is important to include prospective, multicentre scans to create a more robust ML model and to validate our results. Second, aortic segments with high levels of calcification were excluded from the training (baseline) dataset to ensure the ML model was trained on features specifically associated with GCA and atherosclerosis. However, this exclusion could limit the generalizability of the results, as calcifications are commonly observed in clinical GCA populations. Therefore, these segments were not excluded from the follow-up test set, providing a more clinically representative evaluation. Third, this study focused on the aorta, while GCA can affect a broader range of (smaller) arteries. This limitation arises, among others, due to the constraints of segmentation models. However, with advancements in automated segmentation software, which will enable the segmentation of smaller arteries, future studies could incorporate a more comprehensive evaluation of more arteries involved in the GCA disease process [45,46]. Moreover, looking at the patient characteristics, the atherosclerosis group, consisting of patients with type 2 diabetes mellitus, showed significantly higher values for BMI and FBGL compared to the GCA group 1. These higher FBGL values may reduce [^18^F]FDG uptake, affecting intensity features and the model’s performance. Further investigation is needed to explore this relationship in greater detail. Furthermore, we excluded patients from the baseline dataset who received more than three days of (high-dose) prednisolone therapy at the time of the [^18^F]FDG-PET/CT scan to minimise the false negative scans. However, to enhance clinical applicability, training the ML model on [^18^F]FDG-PET scans during prednisolone therapy would be of interest. Finally, the gold standard for follow-up scans (active or inactive GCA) in this study was based on medication changes, which partly depend on the [^18^F]FDG-PET scan assessment by the nuclear medicine physician. This introduces potential bias. However, the ML model outperformed the nuclear medicine physician report in terms of accuracy. We believe our results demonstrate the model’s potential as a decision-making tool, especially because it was tested on challenging follow-up scans. A prospective study, in which nuclear medicine physicians identify active GCA with and without the ML model’s outcome, is necessary to further assess its feasibility.

## 5. Conclusions

To conclude, in this proof-of-concept study, a well performing ML model was developed to identify or exclude GCA activity in follow-up [^18^F]FDG-PET/CT scans. Notably, the performance of the model surpassed that of clinical reports by nuclear medicine physicians. Moreover, an occlusion map was made to visualise the important aortic areas in [^18^F]FDG-PET/CT scans contributing to the outcome of the ML model. However, the validation of the ML model on a larger dataset is needed for it to become a reliable diagnostic tool. Nevertheless, this ML model could become a valuable addition to clinical practise once optimised and validated, providing clinicians an objective tool for identifying GCA activity.

## Figures and Tables

**Table 1:** Patient characteristics of the patients from the baseline set and follow-up set, and PET scan reconstruction parameters. Data shown in *n* (%) or mean ± standard deviation.

Characteristics	GCA—Group 1 (*n* = 34)	Atherosclerosis—Group (*n* = 34)	*p*-Value	GCA—Group 2a (*n* = 7)	GCA—Group 2b (*n* = 12)	*p*-Value
Age (years)	70 ± 8.1	61 ± 8.7	<0.001	68.7 ± 8.6	69.8 ± 8.4	0.799
Sex (female)	20 (59%)	12 (35%)	0.054	7 (100%)	10 (83%)	0.266
BMI (kg/m^2^)	25.9 ± 5.1	31.8 ± 5.6	<0.001	28.1 ± 4.7	29.5 ± 3.7	0.501
FBGL (mmol/L)	6.2 ± 1.3	7.0 ± 1.1	0.003	5.7 ± 0.4	5.6 ± 1.2	0.340
Scanners						
Biograph mCT40	8 (12%)	4 (6%)		4 (21%)	7 (37%)	
Biograph mCT64	26 (38%)	30 (44%)		3 (16%)	5 (26%)	
Kernel						
Gaussian 6.50	26 (38%)	34 (50%)		7 (37%)	11 (58%)	
Gaussian 8.00	8 (12%)	0 (0)		0 (0)	1 (5%)	
Reconstruction method	PSF + TOF 3i21s			PSF + TOF 3i21s		
Slice thickness (mm)	2			2		
Matrix size (pixel × pixel)	256 × 256			256 × 256		

SD = standard deviation; BMI = body mass index; FBGL = fasting blood glucose level; GCA = giant cell arteritis; PSF = point spread function; TOF = time of flight.

**Table 2:** The performance of the final model and the report of the nuclear medicine physicians.

	PPV [95% CI]	NPV [95% CI]	Accuracy [95% CI]
Final model	0.83 [0.44–0.97]	0.85 [0.58–0.96]	0.84 [0.62–0.94]
Clinical report NMP	0.80 [0.38–0.96]	0.79 [0.52–0.92]	0.79 [0.57–0.91]

NMP = nuclear medicine physician; PPV = positive predictive value; NPV = negative predictive value.

## Data Availability

The data that support the findings of this study are available from the corresponding authors upon reasonable request. The data are not publicly available due to privacy or ethical restrictions.

## Supplementary Materials

The following supporting information can be downloaded at: https://www.mdpi.com/article/10.3390/diagnostics15030367/s1, Supplementary part A—Calcification grading, Supplementary part B—technical details texture feature extraction and classification, Supplementary part C—Explainable machine learning model.
