# Supplementary material for: A Machine Learning Model Based on Radiomic Features as a Tool to Identify Active Giant Cell Arteritis on [18F]FDG-PET Images During Follow-Up
Source: Diagnostics (Basel). 2025 Feb 4;15(3):367. doi: 10.3390/diagnostics15030367 (PMC11817507; doi:10.3390/diagnostics15030367)
Supplement: Supplementary file 1 [file diagnostics-15-00367-s001.zip › diagnostics-3428029-supplementary.pdf]

Supplementary part A – Calcification grading

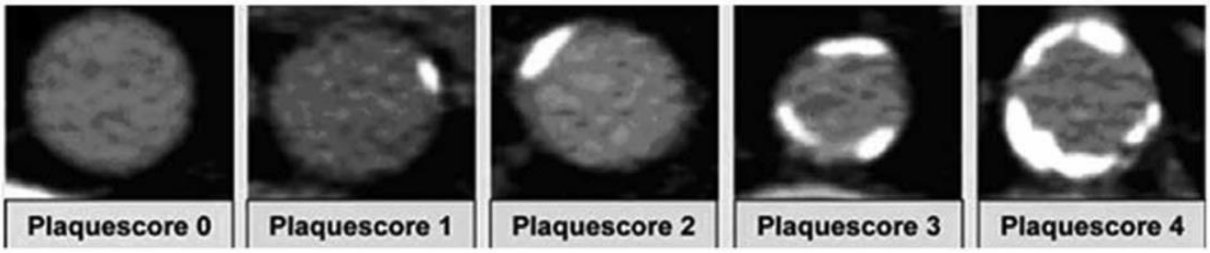

Figure A1. Calcification grading score. Plaque score 0: no calcification, 1: <10%, 2: 10-25%, 3: 25-50%, 4: >50%. This image was adjusted and reprinted from Rominger et al [1].

To ensure a clean dataset for the training of the machine learning model, we excluded aorta segments with a calcification grading score of 3 and 4. So if more then 25% of the aorta was calcified, the segment was excluded from the GCA group 1 (Figure A1).

Supplementary part B – technical details texture feature extraction and classification

For each segment, the SUL was discretized with a fixed bin width of 0.5, defined by equation B1 [2].

$$X_{d,x} = \frac{X_{gl,x} - X_{gl,min}}{W_b} + 1 \qquad X_{d,x} \in Z \qquad \text{(B1)}$$

where $X_{d,x}$ is the resampled intensity of voxel x, $X_{gl,x}$ is the intensity of voxel x before resampling, $X_{gl,min}$ is the lowest intensity value in the VOI and W_b is the bin width. One is added to make the lowest bin value equal to 1 [3]. A total of 95 features were extracted, see below this page the complete list of features.

After feature extraction, the features were standardized using the Z-score. The Z-score is calculated with the following equation:

$$Z = \frac{X - \mu}{\sigma} \qquad \text{(B2)}$$

where X represents the individual feature value, μ the mean of the feature values calculated over the 238 segments of the baseline set and σ the standard deviation of the feature values calculated over all 238 segments. After standardization, features with a high linear correlation (a Pearson correlation coefficient (Pearson's *r*) above 0.9), were removed.

The seven feature selection methods used to perceive the most important features were: least absolute shrinkage and selection operator (LASSO), support vector machines- recursive feature elimination (SVM-RFE), ReliefF, minimum redundancy and maximum relevance ensemble (MRMRe), mutual information (MI), t-score, and ANOVA. To assess the optimal number of features necessary to differentiate between GCA and atherosclerosis, every feature selection method selected nine different number of features (1, 2, 4, 6, 8, 10, 12, 15, and 20 features). That resulted in 9x7 = 63 feature selection models. Seven different classifiers were used to assess the classification quality for each of these 63 ML models: logistic regression (LR), support vector machine-radial basis function kernel (SVM-RBF), random forest (RF), extra tree classifier (ETC), k-nearest neighbour (KNN), extreme gradient boosting (XGBoost), and neural network (NN).

Extracted features

First-order

- 10 Percentile
- 90 Percentile
- Energy
- Entropy
- Interquartile Range
- Kurtosis
- Maximum
- Mean Absolute Deviation
- Mean
- Median
- Minimum
- Range
- Robust Mean Absolute Deviation
- Root Mean Squared

- Skewness
- Total Energy
- Uniformity
- Variance

GLCM

- Autocorrelation
- Cluster Prominence
- Cluster Shade
- Cluster Tendency
- Contrast
- Correlation
- Difference Average
- Difference Entropy
- Difference Variance
- Id
- Idm
- Idmn
- Idn
- Imc1
- Imc2
- Inverse Variance
- Joint Average
- Joint Energy
- Joint Entropy
- MCC
- Maximum Probability
- Sum Average
- Sum Entropy
- Sum Squares

GLDM

- Dependence Entropy
- Dependence Non Uniformity
- Dependence Non Uniformity Normalized
- Dependence Variance
- Gray Level Non Uniformity
- Gray Level Variance
- High Gray Level Emphasis
- Large Dependence Emphasis
- Large Dependence High Gray Level Emphasis
- Large Dependence Low Gray Level Emphasis
- Low Gray Level Emphasis
- Small Dependence Emphasis
- Small Dependence High Gray Level Emphasis
- Small Dependence Low Gray Level Emphasis

GLRLM

- Gray Level Non Uniformity
- Gray Level Non Uniformity Normalized
- Gray Level Variance
- High Gray Level Run Emphasis
- Long Run Emphasis

- Long Run High Gray Level Emphasis
- Long Run Low Gray Level Emphasis
- Low Gray Level Run Emphasis
- Run Entropy
- Run Length Non Uniformity
- Run Length Non Uniformity Normalized
- Run Percentage
- Run Variance
- Short Run Emphasis
- Short Run High Gray Level Emphasis
- Short Run Low Gray Level Emphasis

GLSZM

- Gray Level Non Uniformity
- Gray Level Non Uniformity Normalized
- Gray Level Variance
- High Gray Level Zone Emphasis
- Large Area Emphasis
- Large Area High Gray Level Emphasis
- Large Area Low Gray Level Emphasis
- Low Gray Level Zone Emphasis
- Size Zone Non Uniformity
- Size Zone Non Uniformity Normalized
- Small Area Emphasis
- Small Area High Gray Level Emphasis
- Small Area Low Gray Level Emphasis
- Zone Entropy
- Zone Percentage
- Zone Variance

NGTDM

- Busyness
- Coarseness
- Complexity
- Contrast
- Strength

Quantitative features

- SUL_{mean}
- SUL_{max}

Supplementary part C – Explainable machine learning model

An occlusion sensitivity map of the aorta segments was created using a sliding kernel of zeros of 30x30x15 voxels with a step size of 10x10x5 in x-,y-,z-direction. For every region the change in prediction probability was measured and mapped to visualize the influence per region scaled from -1 to 1.

References

[1] A. Rominger *et al.*, "18F-FDG PET/CT Identifies Patients at Risk for Future Vascular Events in an Otherwise Asymptomatic Cohort with Neoplastic Disease," *Journal of Nuclear Medicine*, vol. 50, no. 10, p. 1611, Oct. 2009, doi: 10.2967/jnumed.109.065151.

[2] R. T. H. Leijenaar *et al.*, "The effect of SUV discretization in quantitative FDG-PET Radiomics: the need for standardized methodology in tumor texture analysis," *Sci Rep*, vol. 5, no. 1, p. 11075, 2015, doi: 10.1038/srep11075.

[3] "IBSI - Image Biomarker Standardisation Initiative," *(Available At Https://Theibsi.Github.Io)*, vol. 0, no. December, 2019, [Online]. Available: https://theibsi.github.io
